# Revisiting *Stygocapitella* (Annelida, Parergodrilidae) in Japan, with insights into their amphi-Pacific diversification

**DOI:** 10.1098/rsos.231782

**Published:** 2024-06-19

**Authors:** Natsumi Hookabe, Naoto Jimi, Shinta Fujimoto, Hiroshi Kajihara

**Affiliations:** ^1^ Research Institute for Global Change (RIGC), JAMSTEC, Yokosuka, Kanagawa 237-0061, Japan; ^2^ Sugashima Marine Biological Laboratory, Graduate School of Science, Nagoya University, Toba, Mie 517-0004, Japan; ^3^ Centre for Marine & Coastal Studies, Universiti Sains Malaysia 11800 USM, Penang, Malaysia; ^4^ Graduate School of Sciences and Technology for Innovation, Yamaguchi University, Yamaguchi 753-8512, Japan; ^5^ Faculty of Science, Hokkaido University, Sapporo 060-0810, Japan

**Keywords:** ghost worm, interstitial, marine invertebrates, meiobenthos, polychaetes

## Abstract

Polychaetes are typically found in marine environments with limited species adapting to semi-terrestrial habitats. The genus *Stygocapitella* comprises interstitial polychaetes dwelling in sandy beach areas around or above the high-water line. Based on molecular data, previous studies suggested the presence of multiple cryptic species in some different localities in the world lumped together as *Stygocapitella subterranea*. In Japan, reports on *Stygocapitella* were scarce, with only one species having been documented 40 years ago at Ishikari Beach in Hokkaido by the name of *S. subterranea*. We revisited these earlier findings and uncovered the presence of two distinct species in *Stygocapitella*. One of these species is herein named *Stygocapitella itoi* sp. nov., while the other corresponds to *S. budaevae*, originally described from the Russian Far East. *Stygocapitella itoi* sp. nov. possesses a chaetal pattern similar to that of *S. australis*, *S. furcata* and *S. pacifica* but can be distinguished from the congeners by two characters: a slightly forked pygidium and forked chaetae consisting of two teeth and two outer prongs. Our multi-locus phylogenetic analysis showed close relationships across the Pacific Ocean in two separated lineages in the genus, suggesting ancient dispersal or allopatric speciation after vicariance events.

## 1. Introduction

Polychaetes are a diverse group of annelid worms that can be found in a wide range of marine ecosystems, encompassing approximately 11 500 species from the world [1]. While the majority of these species are adapted to aquatic environments, there are a few terrestrial species. One of the terrestrial annelid families, Parergodrilidae Reisinger, 1925, comprises two genera: *Parergodrilus* Reisinger, 1925 and *Stygocapitella* Knöllner, 1934. *Parergodrilus* is monotypic, with a single species, *P. heideri* [2], rarely found in leaf litter [3]. *Stygocapitella*, encompassing 11 species, are typically semi-terrestrial and found in the supralittoral zone of sandy beaches [4–6]. Their habitat typically extends vertically from the surface layer to a depth of approximately 1 m, with a preference for sediments that are not excessively wet [7,8]. Species in *Stygocapitella* are characterized by the presence of long, whip-like chaetae in their first chaetiger, but they lack head appendages and parapodia. They are generally slow-moving to the extent that Itô [9] noted that it can be challenging to determine whether they are alive or deceased upon examination.

Before 2017, the genus *Stygocapitella* was represented by a single cosmopolitan species, *S. subterranea* [10], reported from the Baltic Sea, the North Sea, both coastlines of the Atlantic Ocean and the West Pacific Ocean [8,11–13]. This supposedly wide distribution led Giere [5] to assume the existence of multiple cryptic species under the name of *S. subterranea*. Meanwhile, Struck *et al*. [6] designated a neotype for *S. subterrranea*, and described two species, *S. australis* [6] and *S. minuta* [6], from Australia and South Africa, respectively. Subsequently, eight species were described based on morphological and molecular data collected worldwide [4].

In this study, we found two distinct species of *Stygocapitella* at Ishikari Beach, where ‘*S. subterranea*’ was previously reported [11,14]. Based on our morphological examination using scanning electron microscopy (SEM), one of the two species turned out to be new to science. Furthermore, we reconstruct the phylogeny of the genus using a concatenated dataset of multiple genes. The phylogenetic positions of the two Japanese species provide us insights into amphi-Pacific diversification within *Stygocapitella*.

## 2. Material and methods

### 2.1. Sampling and morphological observation

**Figure 1.**
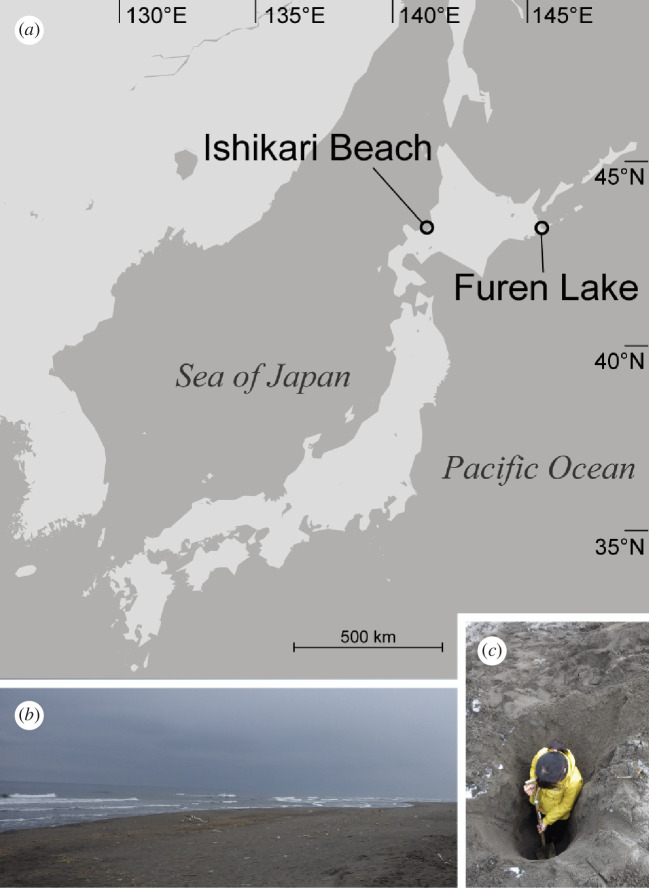
Collection localities of specimens examined in the present study. (*a*) Map showing the collection site of the specimens examined in the present study. (*b*) Lands of the collection site at Ishikari Beach. (*c*) Image of sampling specimens, Ishikari Beach.

All specimens were collected at Ishikari Beach and Furen Lake, Hokkaido, northern Japan, in March and September 2019 (figure 1*a,b*). Pits about 105–180 cm deep were dug with a shovel on the dune, where the groundwater table was about 2 m in depth (figure 1*c*). Specimens were found from pits about 6.5–30 m away from the water line. Sediment samples were scooped at 15–20 cm intervals from the bottom to a depth of 100 cm, brought back to the laboratory, and agitated in tap water to extract animals by freshwater shock. The suspended water was passed through a 250 µm mesh hand net, and the residue was subsequently transferred into seawater. Worms were picked up under a Nikon SMZ 1500 dissecting microscope and photographed with a Nikon D5200 digital camera. For each specimen, the chaetal pattern and the genital organ (therefore, the sex) were examined following Struck *et al*. [6] under an Olympus BX51 compound light microscope with a Nomarski differential interference contrast device before fixation. Specimens for molecular studies were preserved in 99% ethanol. For morphological observation under SEM, specimens were anaesthetized with a MgCl_2_ solution isotonic to seawater and then fixed in 10% seawater-buffered formalin.

For SEM, specimens fixed in 10% seawater-buffered formalin were dehydrated in an ethanol series, critical-point dried in a Hitachi HCP-1, mounted on an aluminium stub, coated with gold in a JEOL JFC-1100 and then examined with a Hitachi S-3000N scanning electron microscope at 15–30 kV accelerating voltage. Type and voucher specimens have been deposited in the National Museum of Science and Technology, Tsukuba (NSMT), Japan.

### 2.2. DNA extraction, polymerase chain reaction amplification and sequencing

Total DNA was extracted using a DNeasy Tissue Kit (Qiagen). DNA fragments were amplified using the primer pairs polyHCO/LCO [15] for partial sequences of the cytochrome *c* oxidase subunit I gene (COI), 16Sar-L/16br-H [16] for 16S rRNA (16S), Stygo_ITS1_F and Stygo_ITS1_R [4] for the internal transcribed spacer region (ITS1) and 1F/9R [17] for 18SrRNA (18S) by an Applied Systems 2720 thermal cycler with a preheating at 94°C for 2 min; 35 cycles of 94°C for 30 s, 50–52°C for 60 s, and 72°C for 60 s; then a final extension at 72°C for 7 min. Nucleotide sequencing was performed using the same primer pairs as for the initial polymerase chain reaction (PCR) amplification and internal primers, 3F/5R [17] and 18Sbi/S2.0 [18] for 18S with an ABI BigDye Terminator v. 3.1 Cycle Sequencing Kit and an ABI 3100 Avant Genetic Analyzer (Applied Biosystems). We determined COI and 16S for all specimens and 18S and ITS1 for two specimens. Sequences obtained in this study have been deposited in DDBJ/EMBL/GenBank.

### 2.3. Phylogenetic analyses

Newly generated sequences were combined with sequences available in GenBank (table 1), and aligned by MAFFT v. 7, employing auto-selected strategy in Geneious Prime v. 2023.2.1 (http://www.geneious.com/). Ambiguous sites were removed using Gblocks v. 0.91b [22], which resulted in COI (596 bp), 16S (418 bp), 18S (1758 bp) and ITS1 (667 bp). To infer the phylogenetic positions of the species examined in this study, maximum-likelihood (ML) analyses were performed using the IQ-TREE software [23]. Best-fit partition models were selected using the IQ-TREE web server [24] as follows: TIM2+F+G4 for the first codon position of COI, TIM3e+G4 for the second codon position of COI, TIM+F+I for the third codon position of COI, TN+F+G4 for 16S, TNe+G4 for 18S and TN+F+I for ITS1. Nodal support values were derived from 1000 ultrafast bootstrap (UFBoot) [23].

**Table 1.** List of species used for phylogenetic analyses in this study with GenBank accession numbers.

species	COI	16S	18S	ITS1	sources
*Leitoscoloplos bifurcatus*	KR781456	KR349351	KR778793	—	Zhadan *et al*. [19]
*Leitoscoloplos fragilis*	FJ612498	AY532341	AY532360	—	Bleidorn [20]; Bleidorn *et al*. [21]
*Stygocapitella americae*_432_10	MN158597	MN164068	MN162914	MN162724	Cerca *et al*. [4]
*Stygocapitella americae*_433_01	MN158590	MN164069	MN162917	MN162720	Cerca *et al*. [4]
*Stygocapitella australis* 392_05	KY503045	—	KY503077	—	Struck *et al*. [6]
*Stygocapitella australis* 393_01	KY503048	—	KY503078	—	Struck *et al*. [6]
*Stygocapitella berniei*_430_01	MN158602	MN164081	MN162921	MN162726	Cerca *et al.* [4]
*Stygocapitella berniei*_430_05	MN158605	MN164084	MN162924	MN162729	Cerca *et al*. [4]
*Stygocapitella budaevae* (Furen Lake)	LC484888	LC484889	—	—	Present study
*Stygocapitella budaevae* (Ishikari Beach)	LC484884	LC484890	LC484886	—	Present study
*Stygocapitella budaevae*_442_20	MN158381	MN164054	MN162903	MN162746	Cerca *et al*. [4]
*Stygocapitella budaevae*_442_22	MN158377	MN164059	MN162906	MN162743	Cerca *et al*. [4]
*Stygocapitella budaevae*_442_6	MN158374	MN164060	MN162912	MN162744	Cerca *et al*. [4]
*Stygocapitella furcata*_432_03	MN158612	MN164343	MN162996	MN162886	Cerca *et al*. [4]
*Stygocapitella furcata*_432_05	MN158613	MN164345	MN162997	MN162887	Cerca *et al*. [4]
*Stygocapitella furcata*_432_06	MN158614	MN164344	MN162998	MN162888	Cerca *et al*. [4]
*Stygocapitella itoi* sp. nov. (Furen Lake)	LC484891	LC484892	—	LC484893	Present study
*Stygocapitella itoi* sp. nov. (Ishikari Beach)	LC484885	LC484894	LC484887	LC484895	Present study
*Stygocapitella josemariobrancoi*_169_09	MN158424	MN164165	MN162973	MN162839	Cerca *et al.* [4]
*Stygocapitella josemariobrancoi*_169_10	MN158392	MN164174	MN162974	MN162825	Cerca *et al*. [4]
*Stygocapitella josemariobrancoi*_169_58	MN158417	MN164164	MN162976	MN162813	Cerca *et al*. [4]
*Stygocapitella josemariobrancoi*_222_04	MN158416	MN164142	MN162984	MN162811	Cerca *et al*. [4]
*Stygocapitella josemariobrancoi*_421_01	MN158471	MN164224	MN162964	MN162852	Cerca *et al.* [4]
*Stygocapitella josemariobrancoi*_422_01	MN158387	MN164135	MN162970	MN162799	Cerca *et al*. [4]
*Stygocapitella josemariobrancoi*_422_02	MN158399	MN164136	MN162971	MN162803	Cerca *et al*. [4]
*Stygocapitella josemariobrancoi*_429_08	MN158429	MN164185	MN162967	MN162851	Cerca *et al.* [4]
*Stygocapitella minuta*_391_17	KY503065	—	KY503075	—	Struck *et al*. [6]
*Stygocapitella minuta*_391_18	KY503066	—	KY503076	—	Struck *et al.* [6]
*Stygocapitella pacifica*_442_10	MN158611	MN164341	MN162994	MN162889	Cerca *et al.* [4]
*Stygocapitella pacifica*_442_11	—	MN164342	MN162995	MN162890	Cerca *et al*. [4]
*Stygocapitella* sp._432_02	MN158382	MN164061	MN162897	MN162736	Cerca *et al*. [4]
*Stygocapitella* sp._432_07	MN158385	MN164063	MN162909	MN162739	Cerca *et al*. [4]
*Stygocapitella subterranea*_227_01	MN158519	MN164285	MN162935	—	Cerca *et al.* [4]
*Stygocapitella subterranea*_320_06	MN158526	MN164298	MN162926	—	Cerca *et al*. [4]
*Stygocapitella subterranea*_320_07	MN158540	MN164315	MN162927	—	Cerca *et al.* [4]
*Stygocapitella subterranea*_321_01	MN158538	MN164300	MN162929	—	Cerca *et al*. [4]
*Stygocapitella subterranea*_321_02	MN158539	MN164301	MN162952	—	Cerca *et al*. [4]
*Stygocapitella subterranea*_396_04	KY503070	MN164327	MN162938	MN162761	Cerca *et al*. [4]
*Stygocapitella subterranea*_396_05	KY503071	MN164313	MN162962	MN162760	Cerca *et al*. [4]
*Stygocapitella subterranea*_403_03	MN158508	MN164265	MN162950	MN162762	Cerca *et al*. [4]
*Stygocapitella subterranea*_403_04	MN158567	MN164266	MN162942	—	Cerca *et al*. [4]
*Stygocapitella westheidei*_426_01	MN158481	MN164233	MN162928	MN162768	Cerca *et al.* [4]
*Stygocapitella westheidei*_427_01	MN158484	MN164259	MN162943	MN162781	Cerca *et al.* [4]
*Stygocapitella westheidei*_428_01	MN158491	MN164263	MN162946	MN162791	Cerca *et al*. [4]
*Stygocapitella westheidei*_428_02	MN158498	MN164247	MN162969	MN162790	Cerca *et al*. [4]
*Stygocapitella westheidei*_428_03	MN158488	MN164248	MN162947	MN162769	Cerca *et al*. [4]
*Stygocapitella zecai*_324_04	MN158587	MN164131	MN162992	MN162876	Cerca *et al.* [4]
*Stygocapitella zecai*_440_01	MN158588	MN164092	MN162986	MN162872	Cerca *et al*. [4]

Uncorrected pairwise genetic distances were calculated based on 657 bp of COI by MEGA v. 7 [25].

## 3. Results

### 3.1. Taxonomy

Family Parergodrilidae Reisinger, 1925

Genus *Stygocapitella* Knöllner, 1934

[Japanese name: Sunaito-gokai]

Type species. *Stygocapitella subterranea* Knöllner, 1934

*Stygocapitella budaevae* Cerca, Meyer, Purschke & Struck, 2020

[New Japanese name: Kita-sunaito-gokai]

Figure 2*a*,*h*

**Figure 2.**
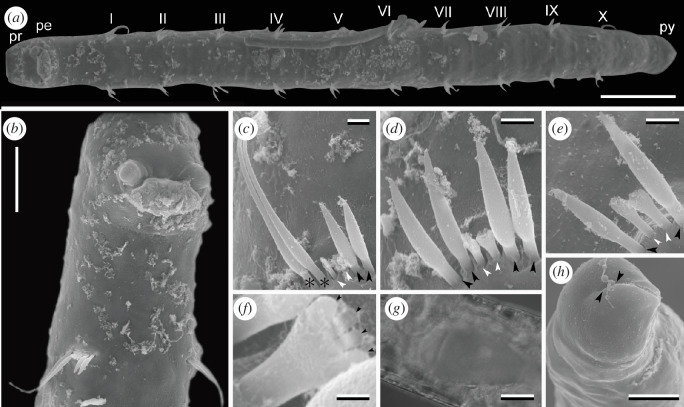
*Stygocapitella budaevae*, female (NSMT ###2). (*a*) Whole body, ventral view; Roman numerals represent the chaetigers. (*b*) Magnification of anterior end, ventrolateral view. (*c*) Chaetiger 1. (*d*) Chaetiger 2. (*e*) Chaetiger 3; asterisks mark whip-like bilimbate chaetae, black arrowheads mark bilimbate chaetae, and white arrowheads mark forked chaetae. (*f*) Magnification of forked chaetae; black arrowheads mark teeth of forked chaetae. (*g*) Oocytes. (*h*) Posterior end showing slightly forked pygidium; arrowheads point to the anal slit on pygidium. (*a–f*), (*h*) SEM images. (*g*) Microphotograph taken under light microscopy. Abbreviations: pe, peristomium; pr, prostomium; py, pygidium. Scale bars: (*a*) 100 µm, (*b*) 50 µm, (*c–e*) 5 µm, (*f*) 1 µm, (*g*, *h*) 25 µm.

#### 3.1.1. Material examined

Five specimens were collected at 27 m inland from a high water line, Ishikari Beach, Hokkaido Prefecture, Japan (43°14.8283′ N, 141°20.8683′ E). NMST-Pol 113496, female, preserved in formalin, 120 cm depth, on 5 March 2019. NMST-Pol 113497, female, Au-coated and mounted on a SEM stub, 120 cm depth, on 5 March 2019. NMST-Pol 113498, female, preserved in formalin, 120 cm depth, on 6 March 2019; NMST-Pol 113499, male, preserved in formalin, 130 cm depth, on 7 March 2019; NMST-Pol 113500, male, preserved in 99% EtOH, 150 cm depth, on 19 March 2019. Two specimens were collected at 6 m inland from a high water line, Furen Lake, Hokkaido Prefecture, Japan (43°17'54.9" N, 145°23'07.7" E). NMST-Pol 113501, male, preserved in 10% formalin, 130 cm depth, on 13 September 2019. NMST-Pol 113502, male, preserved in 10% formalin, 130 cm depth, on 13 September 2019.

#### 3.1.2. Description

Body 0.9–1.0 mm in length, 100 µm in width; whitish and translucent in life. Prostomium broadly rounded, without appendages; peristomium followed by 1 achaetiger + 10 chaetigers + 2 achaetigers (= 13 segments) (figure 2*a,b*). Chaetiger 1 bearing two whip-like bilimbate, two bilimbate and two forked chaetae (figure 2*c*). Chaetiger 2 possessing four bilimbate and two forked chaetae (figure 2*d*), remaining 3–10 chaetigers with two bilimbate and two forked chaetae (figure 2*e*). Forked chaetae comprise two regular teeth between the outer prongs (figure 2*f*). Male with paired spermioducts opening ventrally in chaetiger 9. Female with genital pores at ventral boundary between chaetigers 9 and 10, and possessing one–two oocytes (20–75 µm in length) (figure 2*g*). Pygidium slightly forked (figure 2*h*).

#### 3.1.3. Remarks

The chaetal pattern of chaetiger 1 (two whip-like bilimbate, two bilimbate and two forked chaetae) and the chaetal pattern of chaetiger 2 (four bilimbate and two forked chaetae) agree with the morphology of *S. budaevae* Cerca *et al.* [4].

#### 3.1.4. Distribution and habitat

The species is known from Volchanets, Primorsky Krai region, Russia and Ishikari Beach and Furen Lake, Hokkaido, Japan; beach with medium-sized sand grains at or above the higher water [4].

*Stygocapitella itoi* sp. nov.

[New Japanese name: Ito-sunaito-gokai]

(figure 3*a*–*g*)

**Figure 3.**
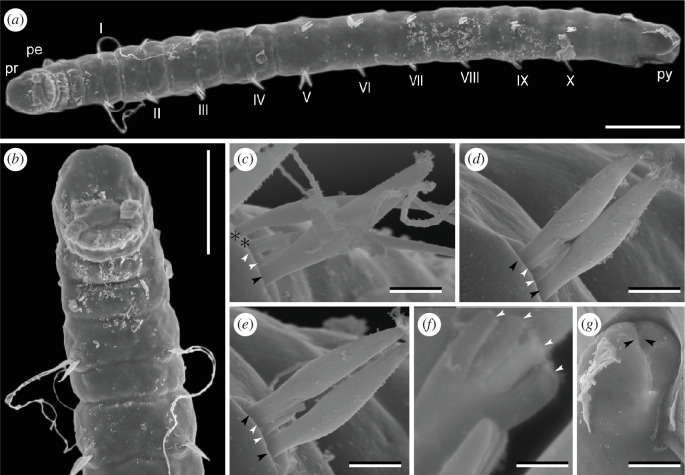
*Stygocapitella itoi* sp. nov., male, paratype (NSMT ###9), SEM images. (*a*) Whole body, ventral view; Roman numerals represent the chaetigers. (*b*) Magnification of anterior end, ventral view. (*c*) Chaetiger 1. (*d*) Chaetiger 2. (*e*) Chaetiger 3; asterisks mark whip-like bilimbate chaetae; black arrowheads mark bilimbate chaetae, and white arrowheads mark forked chaetae. (*f*) Magnification of forked chaetae; white arrowheads mark teeth of forked chaetae. (*g*) Posterior end showing slightly forked pygidium; arrow heads point to anal slit on pygidium. Abbreviations: pe, peristomium; pr, prostomium; py, pygidium. Scale bars: (*a*, *b*) 100 µm, (*c–e*) 5 µm, (*f*) 1 µm, (*g*) 25 µm.

#### 3.1.5. Type materials

Two type specimens, all collected at 27 m inland from the high water line, Ishikari Beach, Hokkaido Prefecture, Japan (43°14.8283′ N, 141°20.8683′ E). Holotype: NMST-Pol H-926, male, preserved in 99% ethanol, 140 cm depth, on 6 March 2019. Paratype: NMST-Pol P-927, male, Au-coated and mounted on a SEM stub, 140 cm depth, on 6 March 2019.

#### 3.1.6. Additional materials

One female specimen was used for DNA extraction, 150 cm depth at the same locality as type materials, on 7 March 2019. One male specimen collected at Furen Lake (43°17'54.9" N, 145°23'07.7" E), 105 cm depth on 13 September 2019, was used for DNA extraction.

#### 3.1.7. Description

Body 0.9–1.2 mm in length, 100 µm in width, whitish and translucent in life. Prostomium broadly rounded, without appendages; peristomium followed by 1 achaetiger + 10 chaetigers + 2 achaetigers (=13 segments) (figure 3*a,b*). Chaetiger 1 was equipped with two whip-like bilimbate, one bilimbate and two forked chaetae (figure 3*c*). Chaetigers 2–10 bearing two bilimbate and two forked chaetae (figure 3*d,e*). Forked chaetae comprise two regular teeth between the outer prongs (figure 3*f*). Male with paired spermioducts opening ventrally in chaetiger 9. Female with genital pores at ventral boundary between chaetigers 9 and 10, oocytes not recognized. Pygidium slightly forked (figure 3*g*).

#### 3.1.8. Remarks

*Stygocapitella itoi* sp. nov. possesses the same chaetal pattern as *S. australis*, *S. furcata* [4] and *S. pacifica* [4] but is distinguishable from the last three by: (i) having a slightly forked pygidium and (ii) having forked chaetae that comprise two teeth and two outer prongs.

#### 3.1.9. Etymology

The new species is named in honour of Dr Tatsunori Itô (1945–1990), who greatly contributed to Japanese meiobenthology through a handbook for the general public, *Organisms in Sand Interstices* [9].

#### 3.1.10. Distribution and habitat

The species is known from Ishikari Beach and Furen Lake, Hokkaido; dunes of sea-coast, moist sand.

### 3.2. Phylogeny and genetic distances

**Figure 4.**
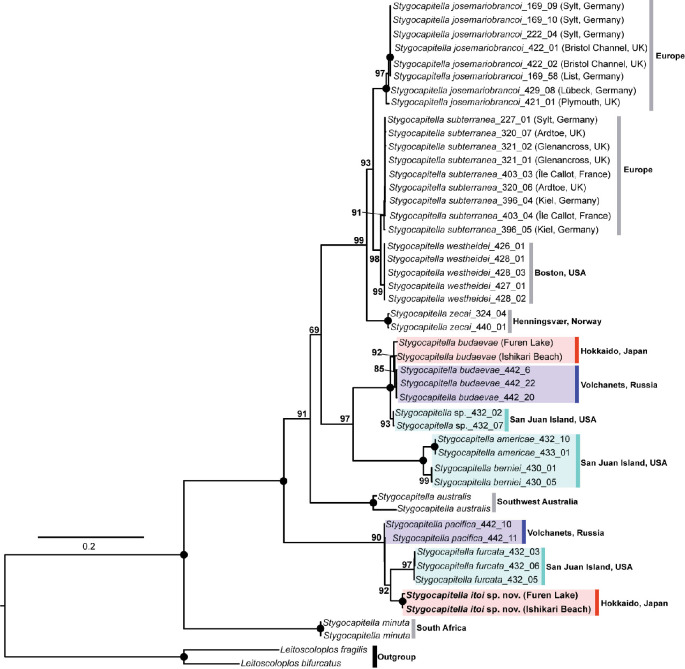
A maximum-likelihood tree of the genus *Stygocapitella* based on concatenated sequences of COI, 16S, 18S and ITS1. Numbers near nodes indicate UFBoot values generated by maximum-likelihood analysis with 1000 replicates in IQ-TREE. Solid circles represent full support values.

In the resulting tree, two specimens of *S. budaevae* collected from Hokkaido, Japan, formed a clade with specimens from Volchanets, Russia (UFBoot = 92%) (figure 4). The *S. budaevae* clade was found to be sister-related with a clade comprising unidentified species from San Juan Island, USA (*Stygocapitella* sp._432_02 and *Stygocapitella* sp._432_07); this relationship was fully supported (figure 4).

The newly described species, *S. itoi* sp. nov., was a sister taxon to *S. furcata* collected from San Juan Island, USA (UFBoot = 92%) (figure 4). This clade was then sister to *S. pacifica* from Volchanets, Russia (UFBoot = 90%)

The COI sequences of specimens from Ishikari Beach and Furen Lake were identical to each other within *S. budaevae* and *S. itoi* sp. nov., respectively. Interspecific genetic distances between Japanese and Russian specimens of *S. budaevae* were 0.12–3.09% in uncorrected *p*-distance, while it was 17.1% between *S. furcata* and *S. itoi* sp. nov.

## 4. Discussion

### 4.1. Revisiting taxonomy of Japanese *Stygocapitella*

In the present study, we identified two species of *Stygocapitella* in Hokkaido, Japan (figures 2 and 3), one of which was herein described as *S. itoi* sp. nov. Our morphological observations support that chaetal patterns are important for distinguishing species in *Stygocapitella* [4]. Furthermore, our study highlights that pygidium shape and the number of teeth in forked chaetae are useful for species distinction. Notably, these additional characteristics enabled the differentiation of *S. itoi* sp. nov. from its closely related species, *S. furcata* (figure 4). In addition to these morphological differences, *S. itoi* sp. nov. can be differentiated from *S. furcata* by COI genetic distances; the values were comparable with interspecific thresholds reported in *Stygocapitella* [4].

The present study represents a re-examination of the earlier reports by Ito [11] of *S. subterranea* from Ishikari Beach. Although we successfully obtained *Stygocapitella* specimens on the beach, we cannot determine whether [9,11] material represented *S. budaevae*, *S. itoi* sp. nov., or neither/both of these species because [9] lacks illustrations of essential characters for distinguishing different *Stygocapitella* species. Moreover, detailed information about the sampling site for [9,11] *Stygocapitella* specimens was not recorded in his work. According to additional notes found in [9], it is reasonable to infer that the sampling location was probably in the vicinity of Ishikari Bay New Port (constructed 1973–1982), approximately 5 km away from our study site. In light of recent global biogeographic analyses by Cerca *et al*. [4] and Itô [9,11], *Stygocapitella* specimens from Ishikari Beach are unlikely to be *S. subterranea*. European populations associated with the name *S. subterranea* are genetically isolated from West Pacific populations, as shown in Cerca *et al*. [4]. Consequently, the previous identification of these specimens as *S. subterranea* should be revised to accurately reflect their taxonomic status as *S. budaevae, S. itoi* sp. nov., or neither of the two species.

### 4.2. Amphi-Pacific diversification in *Stygocapitella*

Our phylogenetic analysis revealed closely related relationships on both sides of the Pacific Ocean, notably between *S. budaevae* and an unidentified species from San Juan Island (represented by *Stygocapitella* sp._432_02 and *Stygocapitella* sp._432_07), and between *S. furcata* and *S. itoi* sp. nov. (figure 4). These relationships correspond to the patterns previously inferred by Cerca *et al*. [4]. Such amphi-Pacific relationships, where closely related species are found on both sides of the Pacific Ocean, have been reported in various terrestrial organisms [26]. Common vicariance events are often suggested as contributing to the diversification of these species [27]. While the exact mechanisms of these dispersal events remain uncertain, particularly given the unclear dispersal capacity of *Stygocapitella*, our findings indicate the potential for ancient lineage dispersal across the Pacific Ocean or allopatric speciation following vicariance events in two separate lineages in *Stygocapitella*.

## Data Availability

Genetic data can be obtained from Genbank (accession nos. LC484884–LC484894: https://www.ncbi.nlm.nih.gov/genbank/).
