# Correction to: ‘Revisiting *Stygocapitella* (Annelida, Parergodrilidae) in Japan, with insights into their amphi-Pacific diversification’ (2024), by Natsumi Hookabe *et al*.

**DOI:** 10.1098/rsos.241162

**Published:** 2024-08-28

**Authors:** Natsumi Hookabe, Naoto Jimi, Shinta Fujimoto, Hiroshi Kajihara

**Affiliations:** ^1^ Research Institute for Global Change (RIGC), JAMSTEC, Yokosuka, Kanagawa 237-0061, Japan; ^2^ Sugashima Marine Biological Laboratory, Graduate School of Science, Nagoya University, Sugashima, Toba, Mie 517-0004, Japan; ^3^ Centre for Marine & Coastal Studies, Universiti Sains Malaysia, Penang 11800, Malaysia; ^4^ Graduate School of Sciences and Technology for Innovation, Graduate School of Sciences and Technology for Innovation, Yamaguchi University, Yamaguchi, Yamaguchi 753-8512, Japan; ^5^ Graduate School/Faculty of Science, Hokkaido University, Sapporo 060-0810, Japan

**Keywords:** ghost worm, interstitial, marine invertebrates, meiobenthos, polychaetes

*R. Soc. Open Sci.* **11**, 231782 (Published online 19 June 2024). (https://doi.org/10.1098/rsos.231782).

The electronic publication of Hookabe *et al.* (2024) [1] does not include evidence of registration in ZooBank (https://zoobank.org/) within the work itself, which is a requirement by Articles 8.5.3 and 78.2.4 of the International Code of Zoological Nomenclature (2012) [2]. As a result, the newly proposed species name, *Stygocapitella itoi*, was rendered unavailable.

To rectify this situation, we have registered this correction article under the ZooBank LSID: urn:lsid:zoobank.org:pub:22DF47ED-12F3-4A2A-8854-454A650689BC along with species descriptions. Furthermore, we corrected a part of ‘Material and Methods’ and figure legends where specimen deposition IDs were incorrectly shown in Hookabe *et al*. [1].

## Material and methods

All specimens were collected at Ishikari Beach and Furen Lake, Hokkaido, northern Japan, in March and September 2019 (figure 1*a,b*). Pits about 105−180 cm deep were dug with a shovel on the dune, where the groundwater table was about 2 m in depth (figure 1*c*). Type and voucher specimens have been deposited in the National Museum of Nature and Science, Tsukuba (NMST), Japan.

**Figure 1.**
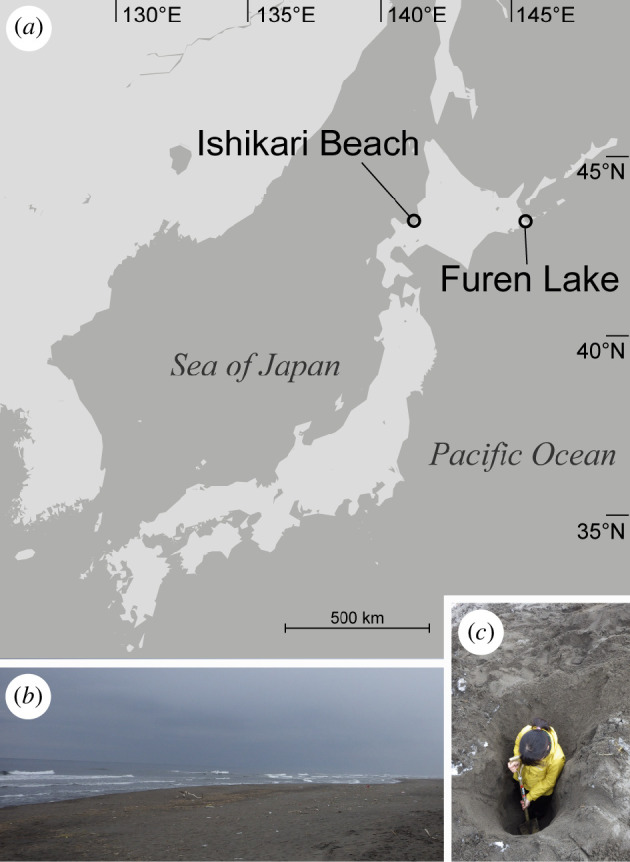
Collection localities of specimens examined in the present study. (*a*) Map showing the collection site of the specimens examined in the present study. (*b*) Lands of the collection site at Ishikari Beach. (*c*) Image of sampling specimens, Ishikari Beach.

## Results

### Taxonomy

Family Parergodrilidae Reisinger 1925 [3]

Genus *Stygocapitella* Knöllner 1934 [4]

[Japanese name: Sunaito-gokai]

Type species. *Stygocapitella subterranea* Knöllner 1934 [4]

***Stygocapitella budaevae*** **Cerca, Meyer, Purschke & Struck 2020**

[New Japanese name: Kita-sunaito-gokai]

(figure 2*a–h*)

**Figure 2.**
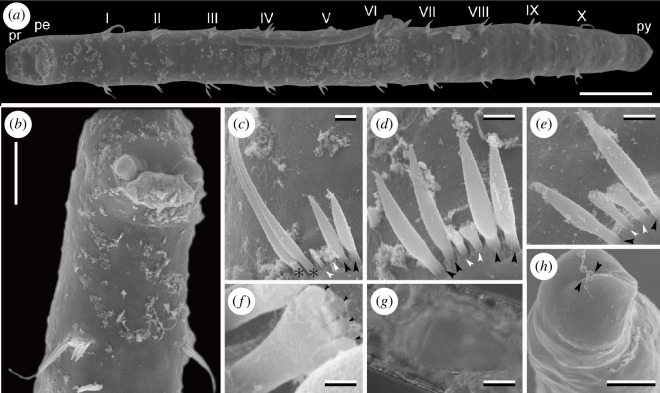
*Stygocapitella budaevae*, female (NMST-Pol 113497). (*a*) Whole body, ventral view; Roman numerals represent the chaetigers. (*b*) Magnification of anterior end, ventrolateral view. (*c*) Chaetiger 1. (*d*) Chaetiger 2. (*e*) Chaetiger 3; asterisks mark whip-like bilimbate chaetae, black arrowheads mark bilimbate chaetae, and white arrowheads mark forked chaetae. (*f*) Magnification of forked chaetae; black arrowheads mark teeth of forked chaetae. (*g*) Oocytes. (*h*) Posterior end showing slightly forked pygidium; arrowheads point to the anal slit on pygidium. (*a*–*f*), (*h*) SEM images. (*g*) Microphotograph taken under light microscopy. Abbreviations: pe, peristomium; pr, prostomium; py, pygidium. Scale bars: (*a*) 100 µm, (*b*) 50 µm, (*c*–*e*) 5 µm, (*f*) 1 µm, (*g,h*) 25 µm.

### Material examined

Five specimens were collected at 27 m inland from a high water line, Ishikari Beach, Hokkaido Prefecture, Japan (43°14.8283ʹ N, 141°20.8683ʹ E). NMST-Pol 113496, female, preserved in formalin, 120 cm depth, on 5 March 2019. NMST-Pol 113497, female, Au-coated and mounted on a SEM stub, 120 cm depth, on 5 March 2019. NMST-Pol 113498, female, preserved in formalin, 120 cm depth, on 6 March 2019; NMST-Pol 113499, male, preserved in formalin, 130 cm depth, on 7 March 2019; NMST-Pol 113500, male, preserved in 99% EtOH, 150 cm depth, on 19 March 2019. Two specimens were collected at 6 m inland from a high water line, Furen Lake, Hokkaido Prefecture, Japan (43°17ʹ54.9ʺ N, 145°23ʹ07.7ʺ E). NMST-Pol 113501, male, preserved in 10% formalin, 130 cm depth, on 13 September 2019. NMST-Pol 113502, male, preserved in 10% formalin, 130 cm depth, on 13 September 2019.

### Description

Body 0.9−1.0 mm in length, 100 µm in width; whitish and translucent in life. Prostomium broadly rounded, without appendages; peristomium followed by 1 achaetiger + 10 chaetigers + 2 achaetigers (= 13 segments) (figure 2*a,b*). Chaetiger 1 bearing two whip-like bilimbate, two bilimbate and two forked chaetae (figure 2*c*). Chaetiger 2 possessing four bilimbate and two forked chaetae (figure 2*d*), remaining 3−10 chaetigers with two bilimbate and two forked chaetae (figure 2*e*). Forked chaetae comprise two regular teeth between the outer prongs (figure 2*f*). Male with paired spermioducts opening ventrally in chaetiger 9. Female with genital pores at ventral boundary between chaetigers 9 and 10, and possessing 1–2 oocytes (20−75 µm in length) (figure 2*g*). Pygidium slightly forked (figure 2*h*).

### Remarks

The chaetal pattern of chaetiger 1 (two whip-like bilimbate, two bilimbate and two forked chaetae) and the chaetal pattern of chaetiger 2 (four bilimbate and two forked chaetae) agree with the morphology of *S. budaevae* Cerca, Meyer, Purschke & Struck 2020 [5].

### Distribution and habitat

The species is known from Volchanets, Primorsky Krai region, Russia and Ishikari Beach and Furen Lake, Hokkaido, Japan; beach with medium-sized sand grains at or above the higher water [5].

***Stygocapitella itoi*** **sp. nov.**

[New Japanese name: Ito-sunaito-gokai]

(figure 3*a–g*)

**Figure 3.**
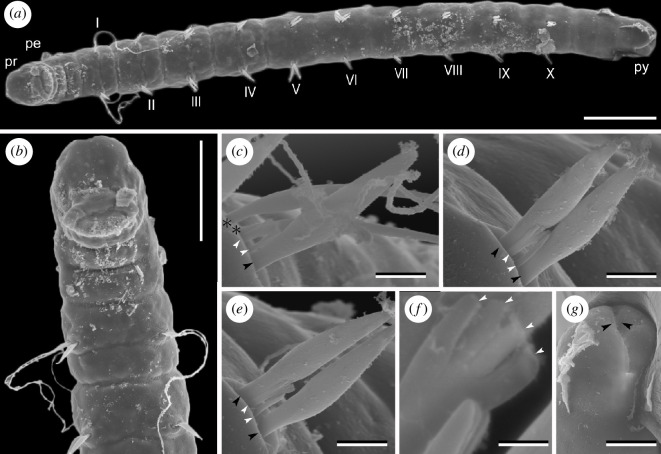
*Stygocapitella itoi* sp. nov., male, paratype (NMST-Pol P−927), SEM images. (*a*) Whole body, ventral view; Roman numerals represent the chaetigers. (*b*) Magnification of anterior end, ventral view. (*c*) Chaetiger 1. (*d*) Chaetiger 2. (*e*) Chaetiger 3; asterisks mark whip-like bilimbate chaetae; black arrowheads mark bilimbate chaetae, and white arrowheads mark forked chaetae. (*f*) Magnification of forked chaetae; white arrowheads mark teeth of forked chaetae. (*g*) Posterior end showing slightly forked pygidium; arrow heads point to anal slit on pygidium. Abbreviations: pe, peristomium; pr, prostomium; py, pygidium. Scale bars: (*a,b*) 100 µm, (*c*–*e*) 5 µm, (*f*) 1 µm, (*g*) 25 µm.

### ZooBank

urn:lsid:zoobank.org:act:E783F15C−1ED9−49B8-BCF2−3A25EEE7E421.

### Type materials

Two type specimens, all collected at 27 m inland from the high water line, Ishikari Beach, Hokkaido Prefecture, Japan (43°14.8283′ N, 141°20.8683′ E). Holotype: NMST-Pol H−926, male, preserved in 99% ethanol, 140 cm depth, on 6 March 2019. Paratype: NMST-Pol P−927, male, Au-coated and mounted on a SEM stub, 140 cm depth, on 6 March 2019.

### Additional materials

One female specimen used for DNA extraction, 150 cm depth at the same locality as type materials, on 7 March 2019. One male specimen collected at Furen Lake (43°17ʹ54.9ʺ N, 145°23ʹ07.7ʺE), 105 cm depth on 13 September 2019, used for DNA extraction.

### Description

Body 0.9−1.2 mm in length, 100 µm in width, whitish and translucent in life. Prostomium broadly rounded, without appendages; peristomium followed by 1 achaetiger + 10 chaetigers + 2 achaetigers (= 13 segments) (figure 3*a,b*). Chaetiger 1 was equipped with two whip-like bilimbate, one bilimbate and two forked chaetae (figure 3*c*). Chaetigers 2−10 bearing two bilimbate and two forked chaetae (figure 3*d,e*). Forked chaetae comprise two regular teeth between the outer prongs (figure 3*f*). Male with paired spermioducts opening ventrally in chaetiger 9. Female with genital pores at ventral boundary between chaetigers 9 and 10, oocytes not recognized. Pygidium slightly forked (figure 3*g*).

### Remarks

*Stygocapitella itoi* sp. nov. possesses the same chaetal pattern as *Stygocapitella australis*, *S. furcata* Cerca, Meyer, Purschke & Struck 2020, and *S. pacifica*, but distinguishable from the latter three by (i) having a slightly forked pygidium and (ii) having forked chaetae that comprise two teeth and two outer prongs.

### Etymology

The new species is named in honour of Dr. Tatsunori Itô (1945−1990), who greatly contributed to Japanese meiobenthology by a handbook for the general public *Organisms in Sand Interstices* [6].

### Distribution and habitat

The species is known from Ishikari Beach and Furen Lake, Hokkaido; dunes of seacoast, moist sand.
